# Progress in Surgery

**Published:** 1899-07-08

**Authors:** 


### PROGRESS IN SURGERY.

### SURGERY OF THE LIVER AND GALL BLADDER.

**Cirrhosis of the Liver.**—There is an interesting and suggestive paper by Dr. Robert Weir on the possibility of re-establishing the portal circulation by surgical operation in cirrhosis of the liver. The suggestion seems to have arisen with Dr. Drummond and Mr. Rutherford Morrison in 1896, who obtained adhesions between the liver and parietes and the omentum and parietes by vigorous rubbing of the viscera with sponges.[1] This included the liver, spleen, and omentum, and the latter was also fixed to the abdominal parietes. In one case the operation failed, in the others it was eminently successful. Following up this idea, Dr. Weir planned a series of experiments on animals to determine if some venous anastomosis could not be effected between the portal vein and the inferior vena cava. Eck, however, had proved the disastrous results of this in animals in 1877, for seven out of eight animals died within a week of the operation.

Hahn experimented in 1892, and unexpectedly found that the portal blood introduced directly into the systemic circulation in this way acted as a poison, causing nervous irritation, convulsions, and coma.

Weir performed the following operation: A four-inch vertical incision was made on the right side of the upper third of the rectus muscle. The liver, diaphragm, and parietal peritoneum were freely scraped, and the omentum stitched on either side of the wound. The pelvis was drained. The patient died on the fifth day, the result of a general peritonitis. This probably arose by infection from the drainage tube.

The total results then of the operation are that, of seven published cases, two recovered, two were not benefited, and three died—one from shock and two from sepsis. Thus, although the procedure may work wonders in a few cases, it is perhaps hazardous in the majority.

**Resection of the Liver.**—Forty cases of hepatic tumour have been collected in which laparotomy was performed. Half the cases were malignant; a quarter of them were syphilitic. Death resulted in one-sixth of the cases, chiefly from hæmorrhage and from sepsis. Of malignant cases recurrence is the rule if the patient survives the operation. Mayo Robson has reported three cases. One died soon after the operation, and two died within a few months from recurrence.[2] Although cases may be apparently malignant Robson advocates operation, for it occasionally happens that the condition turns out to be inflammatory even if it appear malignant when explored by operation. He evidently regards gallstones as a cause of cancer, and thinks that earlier operation on the gall bladder would diminish the mortality from cancer. If there be any secondary malignant troubles in the liver or adjoining viscera, nothing more than simple exploration should be done. In a case recently reported by Hochenegg, of Vienna, a patient died three years after removal of the gall bladder and adjoining part of the liver for cancer.

The operative treatment of gummata of the liver is illustrated by three cases described by Mr. W. G. Spencer, and by one detailed by Mr. Rushton Parker. In the last-named a portion only was removed, the immediate symptoms being relieved, and a rapid recovery following with antisyphilitic treatment.

**Hydatid Cysts.**—Hydatid cysts of the liver are always of interest. Mr. Betham Robinson has recently described a case in which hydatids of the liver and lung were successfully removed.[3] The hydatid of the liver was first removed. Then a skiagram was taken of the dull upper area of the right lung, which proved to be opaque to the X rays. This part was explored and a hydatid cyst enucleated about the size of an orange. A drainage tube was inserted. A case of the disease, in which the daughter cysts were discharged by the common bile duct, is given by Dr. Henry Berg.[4] The liver was very large, and after attacks of colicky pain the patient passed daughter cysts in the stools. A series of cases have been presented by Dr. Robert Ritchie in the *Australasian Medical Journal,* including hydatids of the lung, blockage of the common bile duct, and a hydatid cyst behind the sterno-mastoid.

**Operation on the Gall Tracts.**—American surgeons are imbued with the idea of air distension of hollow parts, probably from the success of Howard Kelly's inflation procedures. Weller Van Hook has suggested its employment in operations upon the gall tracts. To facilitate this he has had an instrument made which consists essentially of a discoid nozzle, which is passed into an opening in the gall bladder, and through which the gall tracts are inflated. When inflation is practised the air acts as a probe, and finds its way quickly into the passages. The advantages claimed are that it makes the identification of the gall-ducts an easy matter, and renders easy the location of any obstruction. It will indicate perforations, diverticula, &c. It facilitates the passage of a probe. It is a sure test for the accuracy of suturing of the ducts.

As with renal calculi, so with those of the gall-bladder —delay in operation is fraught with danger of after-complications. To emphasise this point Gilbert Barling has brought forward a few cases in the *Birmingham Medical Review* for last December.

[1] New York Med. Rec., Feb. 4, 1899. [2] Brit. Med. Jour., Oct. 29, 1898. [3] Brit. Med. Jour., Mar. 18, 1899. [4] Med. Rec., Jan. 7, 1899.

